# Sub-chronic exposure to crude acetylene results in the development of deleterious cardio metabolic changes in *Sprague Dawley* rats

**DOI:** 10.1371/journal.pone.0337172

**Published:** 2025-11-26

**Authors:** Caroline Gatwiri Gitonga, Charles Githinji, Boniface Chege, Frederick Bukachi, Peter Waweru

**Affiliations:** 1 Department of Human Anatomy and Medical Physiology, Faculty of Health Sciences, University of Nairobi, Nairobi, Kenya; 2 Dedan Kimathi University of Science and Technology, School of Health Sciences, Nyeri, Kenya; Atma Ram Sanatan Dharma College University of Delhi, INDIA

## Abstract

**Objective:**

Calcium carbide-derived acetylene is widely used as an artificial fruit ripening agent despite its potential health risks. This study aimed to investigate the effects of sub-chronic exposure to crude acetylene on cardiometabolic parameters using a rodent model.

**Methods:**

Twenty-four male Sprague Dawley rats were randomized into four groups: control (no exposure) and three test groups exposed to 58,000 ppm crude acetylene for 10, 30, or 60 minutes daily over 42 days. Body weight, fasting blood glucose, oral glucose tolerance, hepatic triglyceride levels, adipose tissue mass, liver enzyme activity, and oxidative stress markers were assessed. Histopathological analysis of liver tissue was also conducted.

**Results:**

Acetylene exposure did not significantly alter body weight but led to dose-dependent increases in central adiposity, hepatic triglycerides, and markers of oxidative stress. Higher doses were also associated with impaired glycemic control, elevated liver enzyme levels, and increased free heme concentration in plasma, suggesting oxidative damage and hemolysis. Histological analysis revealed central vein congestion and hepatic structural alterations in exposed groups.

**Conclusion:**

Sub-chronic inhalation of crude acetylene induced metabolic dysfunction characterized by impaired glucose regulation, hepatic steatosis, and oxidative stress, despite no changes in overall body weight. These findings highlight the potential health risks associated with acetylene exposure and underscore the need for regulatory measures to limit its use in fruit ripening.

## Introduction

Commercial Calcium carbide (CaC2), which produces acetylene when dissolved in water that is then utilized in welding, has increasingly been repurposed as a fruit ripening agent [1,2]. This practice remains widespread despite the associated health risks because of its low cost, easy accessibility and simplicity of use compared to the recommended ripening product ethylene [3,4].

The increase in the global demand for fruits which has been accompanied by the globalization of agricultural supply chains has accelerated the adoption of unsafe crop husbandry practices of which artificial ripening using calcium carbide is one, in an attempt to maximize profits [4].

While several studies have investigated the effects of ingesting fruits ripened with calcium carbide, there is a notable lack of research on the effects of chronic inhalational exposure to crude acetylene. This is particularly concerning given its common use in poorly ventilated environments such as informal market settings in developing countries, where handlers and nearby individuals may be exposed through inhalation during the ripening process. This occupational route of exposure is often overlooked compared to ingestion yet it may pose an equal or greater health risk because of the gaseous impurities in crude acetylene.Therefore, focusing on inhalation exposure in this study provides an important complement to ingestion-based research and directly addresses risks faced by these fruit handlers.There are numerous documented effects of both crude and purified acetylene inhalation on physiological systems, many of which are mediated by toxic contaminants present in crude acetylene. Impurities such as phosphine, arsine, and hydrogen sulfide can induce toxic symptoms even at sub-asphyxiating concentrations, potentially leading to systemic effects well before oxygen deprivation occurs [5–7]. Several studies have demonstrated that inhalation of crude acetylene results in elevated serum levels of alanine aminotransferase (ALT) and aspartate aminotransferase (AST), which are established biomarkers of hepatic and cardiac injury, respectively [8]. In a clinical report of an attempted suicide involving industrial acetylene inhalation, the patient presented with hyperglycemia, glycosuria, and ketonuria—despite no prior history of diabetes—mimicking features of diabetic ketoacidosis and suggesting a potential disruption of glucose homeostasis following acetylene exposure [9]. Furthermore, chronic exposure to crude acetylene has been shown to induce dyslipidemia, a key component of metabolic syndrome and a known risk factor for cardiovascular disease [10]. These findings collectively support the rationale for evaluating parameters such as fasting blood glucose, oral glucose tolerance, hepatic triglyceride levels, adiposity, liver enzymes and oxidative in this study.

## Materials and methods

### Study animals

Twenty-four freshly weaned male Sprague Dawley rats weighing between 120g and 140g were randomized into the control, low dose test (exposure to 58000 ppm of crude acetylene for 10 minutes), medium dose test (exposure to 58000 ppm of crude acetylene for 30 minutes) and high dose test (exposure to 58000 ppm of crude acetylene for 60 minutes) experimental groups respectively.The exposure durations were designed to model a gradient of real-life human scenarios: 10 minutes represents brief incidental exposure (e.g., household fruit handling), 30 minutes reflects moderate occupational contact (e.g., market vendors), and 60 minutes simulates prolonged exposure experienced by individuals working in enclosed unregulated ripening facilities. The number of rats (n = 6 per group) was selected to provide sufficient statistical reliability while minimizing animal use, in accordance with the Federation of European Laboratory Animal Science Associations (FELASA) guidelines. They were group housed in standard laboratory animal cages and had *ad libitum* access to standard rat chow (Unga Feeds Ltd. Kenya) and tap water. The following standard laboratory conditions were maintained in the animal house located within the Division of Medical Physiology University of Nairobi: A 12-hour light-dark cycle, room temperature of 20°C, Relative humidity of 15%. The experimental animals were habituated to the experimenter and the experimental environment for seven (7) days prior to the start of the experiment.

### Protocol of exposure to crude acetylene

A sixteen (16) Liter glass inhalation chamber, measuring 25 cm x 25 cm x26 cm and equipped with two outlets on opposite sides, was used as a gas chamber for exposing the experimental animals to acetylene gas. The acetylene gas introduced into the sealed 16 L exposure chamber via a delivery tube. The gas was generated by reacting 2.4 g of freshly ground calcium carbide with 100 ml of water. The volume of acetylene released from this reaction under Standard Temperature and Pressure (STP) conditions was adjusted to its equivalent value at 300 K, which was the internal temperature of the inhalation chamber, and expressed in parts per million cc (ppm). The resulting concentration of the acetylene according to method by Okolie et al. [8] was 58000 ppm. While this value exceeds established occupational exposure limits (~2,500 ppm), it was intentionally selected to mimic poorly ventilated, unregulated informal environments where crude acetylene is used to ripen fruits in bulk. In such settings, particularly in developing countries where enforcement of occupational safety regulations is limited, direct inhalation of crude acetylene by fruit handlers or nearby residents is a realistic possibility. The calcium carbide and water were replaced after every ten (10) minutes to replenish the reactants and ensure continuous production of acetylene throughout the entire exposure period. In addition, the flow meter was adjusted to ensure constant concentration of acetylene in the chamber throughout the duration of exposure so as to effectively mimic the continuous acetylene exposure conditions observed during artificial fruit ripening.

The experimental animals were exposed to acetylene at 24-hour intervals for forty-two (42) days. Control animals underwent the same procedures but without exposure to acetylene. Following each exposure session, the animals were returned to their respective cages.

### Experimental parameter determination

#### Body Weight.

Weekly measurements of body weight (in grams) were determined using a standard laboratory weighing scale and recorded.

#### Glycemic control experiments.

Fasting blood glucose levels were determined measured weekly using the protocol described by Ayala et al. [11]. Briefly, blood samples were obtained through lateral tail vein blood sampling with topical lidocaine applied ten minutes prior to the procedure to minimize pain and reduce stress [12] and the blood glucose determined using Anycall® glucometer and strips.

The Oral Glucose Tolerance Test (OGTT) was conducted on days 14,28 and 42 of the study using the protocol described by Ayala et al [9]following overnight fasting. Briefly, a baseline measurement of blood sugar levels from the lateral tail vein was determined as previously described. A loading dose of glucose (2g/kg of body weight) was then administered to the respective rats via oral gavage. Blood glucose levels were then determined at 30, 60, 90, and 120 minutes after the administration of the glucose loading dose.

#### Euthanasia of experimental animals.

The animals were euthanized on day 42 in their home cage to minimize stress using 100% carbon dioxide gas and immediate harvest of critical organs commenced [13].

#### Adipose tissue depots weight determination.

The adipose tissue depots around the heart, kidney and the peritoneum were carefully dissected and weighed.

### Biochemical tests

#### Hepatic triglycerides.

Livers were harvested from respective animals after euthanization and stored at −80°C until further processing. The hepatic triglycerides levels were assayed using the protocol described in Chege et al. [14]. Briefly, a two (2) grams portion of the respective liver sample was homogenized in 10 ml of phosphate buffer. Two milliliters of the resulting homogenate were then added to four (4) grams of activated charcoal that had been pre-moistened with two (2) milliliters of chloroform to make a paste after which twenty (20) milliliters of chloroform were then added to the paste and the resulting mixture shaken for fifteen (15) minutes and filtered using Whatman’s® filter paper. The resulting filtrate was added to three test tubes that contained equivalent volumes. Additionally, one (1) milliliter of 1% standard corn oil solution was pipetted into three additional test tubes. The excess chloroform was then evaporated by placing all the test tubes in a water bath at 80°C for fifteen (15) minutes. 0.5 mls of alcoholic potassium hydroxide were added to the first and second tube, while 0.5 mls of 95% ethanol were added to the third tube containing the filtrate. The same procedure was repeated for the test tubes containing the standard corn oil solution. The test tubes were then placed in a water bath at 60°C for twenty (20) minutes after which 0.5 ml of 0.2N sulphuric acid were added to each tube, followed by heating in a water bath at 100°C for another twenty (20) minutes. The tubes were then cooled, and 0.1 ml of sodium metaperiodate added to each tube, followed by 0.1 ml of sodium arsenite ten (10) minutes later. Finally, 5 ml of chromotropic acid were added to each tube, and the tubes were kept in a water bath at 100°C for thirty (30) minutes. The optical densities of the solutions at a wavelength of 540 nm were then determined using a spectrophotometer (UV Mini 1240 UV-vis Spectrophotometer, Shimadzu, USA). These optical densities were then utilized to calculate hepatic triglyceride levels using the specific formula:


$$\text{R}=\frac{OPTICAL\;DENSITY\;(O.D)\;SAPONIFIED\;UNKNOWN-O.D.UNSAPONIFIED\;UNKNOWN}{O.D.SAPONIFIED\;CORN\;OIL\;STANDARD-O.D.UNSAPONIFIED\;CORN\;OIL\;STANDARD}$$

Where A = volume of aliquot of chloroform extract in ml. Triglyceride contents in milligram per gram of tissue will then be Triglyceride = 200/A × R × 0.05 = 10R/A.

#### Blood sample collection and liver function tests.

Blood samples were drawn from the euthanized animals via cardiac puncture. Part of the collected samples were used for liver function tests. The gamma-glutamyl transferase (GGT), aspartate aminotransferase (AST), alkaline phosphatase (ALP) and alanine aminotransferase (ALT) activities were then assayed using a biochemistry analyzer located at the Department of Human Pathology, University of Nairobi.

The rest of the respective blood samples were then centrifuged at 1,500G for fifteen (15) minutes to separate the plasma, which was carefully collected and stored at −80°C until analysis.

#### Determination of blood reductive capacity.

The reductive capacity of the plasma samples collected from each experimental animal after euthanization was determined using the nitrocellulose redox permanganometry (NRP) protocol described in Homolak et al [15]. Briefly, 1 μl of plasma from the respective samples were pipetted onto a clean sheet of nitrocellulose membrane and allowed to air-dry completely. The dried samples on the nitrocellulose membrane were then immersed in a potassium permanganate solution (63.27 mM in distilled water) for thirty (30) seconds after which the membrane was rinsed in distilled water to terminate the reaction and enhance contrast, and then left to dry at room temperature. The dried membrane was then digitalized using a high-resolution scanner. The integrated density of the precipitated manganese dioxide spots was analyzed using ImageJ® software (Fiji) with the gel analyzer tool. The resulting data were then statistically analyzed to compare the reductive capacities between the groups.

#### Determination of plasma free heme levels.

The Pyridine Hemochromagen Assay method was used to determine the levels of free heme in plasma, using the protocol described in Barr & Guo, (2015) was employed [16]. Briefly, Solution I was prepared by mixing 2/5 volume of 0.5 M NaOH with 2/5 volume pyridine, diluting to the final volume with deionized water and then adding 1/200 volume of 0.1 M potassium ferricyanide. Solution II (0.1 M potassium ferricyanide) and Solution III (0.5 M sodium dithionite in 0.5 M NaOH) were also freshly prepared. For the assay, 0.5 ml of Solution I was added to 0.5 ml of each plasma sample in a cuvette and mixed well to create the oxidized sample which was then scanned to obtain the initial spectrum at a data interval of 1 nm between 500 and 600 nm, spectral bandwidth of ≤ 2 nm in a spectrophotometer which had utilized Solution I as the blank. 10 μl of Solution III were then added to the oxidized sample, mixed, and scanned immediately and after every minute until the absorbance peak stabilized, indicating the reduced spectrum. The heme concentration was then calculated using the extinction coefficient for reduced pyridine hemochromagen (34.7 mM^-1^ cm^-1^ at 557 nm) and Beer’s Law (A = εcl).

#### Histopathological examination.

After completing the functional studies, the livers were fixed in 10% formalin for three (3) days. Sections were prepared and deparaffinized by immersing them in xylene for 10-15 minutes, followed by rehydration through a series of decreasing alcohol concentrations until water. They were then be placed in Hematoxylin Harris solution 8-10 minutes, rinsed in water and differentiated in 1% acid alcohol solution for 10 seconds and rinsed in tap water again. They then underwent bluing with a solution containing Sodium bicarbonate, MgSO4, and a saturated solution of Lithium carbonate. Counterstaining with 2% aqueous Eosin for 1-3 minutes followed and the sections were then rinsed in tap water with subsequent dehydration through a series of ascending alcohol concentrations and final clearing in xylene. The sections were then dried by pressing on a filter paper. Slides were examined under light microscopy and images captured.

### Ethical approval

All the procedures used in this study followed the guidelines set out in the National Institutes Guide for the Use and Care of Laboratory animals in Research [17]. In addition the procedures complied with the 4Rs Principles of animal research: replacement, reduction, refinement, and responsibility [18].

Approval to conduct this study was sought and obtained from the Biosafety, Animal Use and Ethics Committee, Faculty of Veterinary Medicine, University of Nairobi(REF: FVM BAUEC/2024/568).

### Statistical analysis

Data collected was expressed as Mean ± Standard Error of the Mean (S.E.M.). The data were analyzed using one-way ANOVA using GraphPad Prism 9.1 (GraphPad, USA) with Post-hoc statistical analysis being conducted using the Tukey’s multiple comparisons test in cases of significance (set at p < 0.05).

## Results

### Baseline characteristics

There were no statistically significant differences in the body weights between the 4 groups at the start of the study, (126.2 ± 10.440 g (control) vs. 134.2 ± 8.761 g (low dose test) vs. 123.3 ± 9.186 g (moderate dose test) vs. 129.5 ± 15.99 g (high dose test): F (3, 20) =0.2034: p = 0.9187).

### Effects on body mass

There were no statistically significant differences in body mass among the 4 groups on days 7, 14, 21, 28, 35 and 42.

### Effect on fasting blood glucose

There were no significant differences in the fasting blood glucose between the four groups [4.917 ± 0.1759 mmol/l (control) vs. 5.017 ± 0.2040 (low dose test) vs. 4.817 ± 0.1537 mmol/l (moderate dose test) vs. 4.883 ± 0.1515 mmol/l (high dose test): F (3, 20) = 0.0928: p = 0.8722].

Day 7: There were significant differences in the fasting blood glucose between the four experimental groups on [4.717 ± 0.2023 mmol/l (control) vs. 5.167 ± 0.1838 mmol/l (low dose test) vs. 5.350 ± 0.0992 mmol/l (moderate dose test) vs. 5.367 ± 0.0615 mmol/l (high dose test): F (3, 20) = 1.500: p = 0.0194].

Post-hoc statistical analysis using the Tukey’s multiple comparisons test revealed that there were significant differences between the control group and the high dose test group (p = 0.0270), as well as between the control group and the moderate dose test groups (p = 0.0320).

Day 14: There were no significant differences in the fasting blood glucose between the four groups [4.867 ± 0.1874 mmol/l (control) vs. 5.700 ± 0.4919 (low dose test) vs. 5.667 ± 0.0667 mmol/l (moderate dose test) vs. 5.867 ± 0.1909 mmol/l (high dose test): F (3, 20) = 0.8573: p = 0.0872]

Day 21: There were significant differences in the fasting blood glucose between the four experimental groups on [4.783 ± 0.0980 mmol/l (control) vs. 5.500 ± 0.0730 mmol/l (low dose test) vs. 5.633 ± 0.00882 mmol/l (moderate dose test) vs. 5.883 ± 0.1138 mmol/l (high dose test): F (3, 20) = 0.1455: p < 0.0001].

Post-hoc statistical analysis using Tukey’s multiple comparisons test revealed that there were significant differences between the control group and the high dose test group (p < 0.0001), between the control and the moderate dose test groups (p < 0.0001), between the control and the low dose test groups (P = 0.0002), as well as between the low dose test and high dose test groups (P = 0.0432).

Day 28: There were significant differences in the fasting blood glucose between the four experimental groups [4.867 ± 0.1145 mmol/l (control) vs. 5.417 ± 0.1470 mmol/l (low dose test) vs. 5.683 ± 0.1078 mmol/l (moderate dose test) vs. 6.067 ± 0.1382 mmol/l (high dose test): F (3, 20) = 0.8426: p < 0.0001].

Post-hoc statistical analysis using Tukey’s multiple comparisons test revealed that there were significant differences between the control and the high dose groups (p < 0.0001), between the control and the moderate dose test groups (p = 0.0011), between the control and the low dose test groups (P = 0.0302), as well as between the low dose test and high dose test groups (P = 0.0090).

Day 35: There were significant differences in the fasting blood glucose between the four experimental groups [4.800 ± 0.1592 mmol/l (control) vs. 5.483 ± 0.0654 mmol/l (low dose test) vs. 5.700 ± 0.0577 mmol/l (moderate dose test) vs. 6.083 ± 0.1352 mmol/l (high dose test): F (3, 20) = 1.935: p < 0.0001].

Post-hoc statistical analysis using Tukey’s multiple comparisons test revealed that there were significant differences between the control and the high dose test groups (p < 0.0001), between the control and the moderate dose test groups (p < 0.0001), between the control and the low dose test groups (P = 0.0020), as well as between the low dose test and high dose test groups (P = 0.0064).

Day 42: There were significant differences in the fasting blood glucose between the four experimental groups on [4.400 ± 0.1000 mmol/l (control) vs. 5.583 ± 0.0703 mmol/l (low dose test) vs. 5.950 ± 0.1057 mmol/l (moderate dose test) vs. 6.333 ± 0.1054 mmol/l (high dose test): F (3, 20) = 0.1272: p < 0.0001].

**Fig 1:**
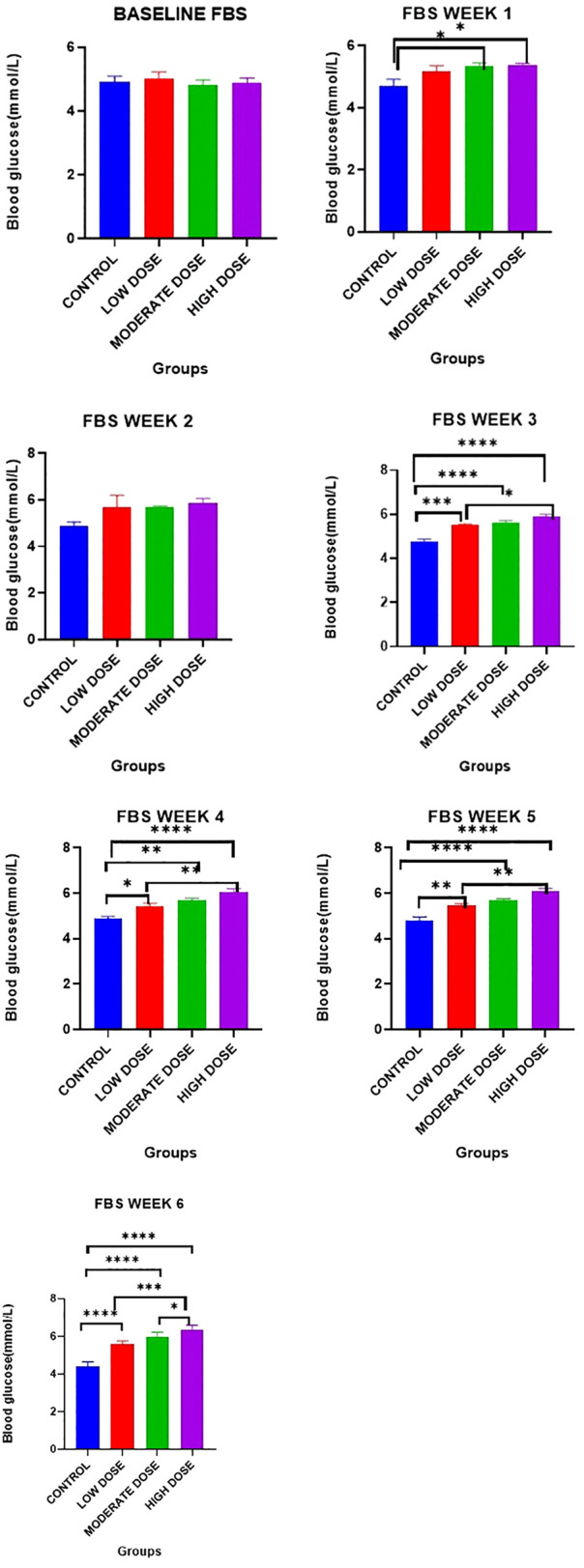
Fasting blood glucose levels (mmol/L) at weekly intervals during the experimental period. Results are expressed As Mean ± SEM. (* P < 0.05, ** P < 0.01, *** P < 0.001, **** P < 0.0001).

Post-hoc statistical analysis using Tukey’s multiple comparisons test revealed that there were significant differences between the control and the high dose test groups (p < 0.0001), between the control and the moderate dose test groups (p < 0.0001), between the control and the low dose test groups (P < 0.0001), between the low dose test and high dose test groups (P = 0.0001), as well as between the moderate dose test and high dose test groups (P = 0.0489). The experimental data is illustrated in Fig 1.

### Oral glucose tolerance

Day 14: There were no significant differences in the mean area under curve (AUC) between the four experimental groups: [699.5 ± 20.35 mmol/L.min (control) vs. 791.3 ± 63.21 mmol/L.min (low dose test) vs. 827.8 ± 25.69 mmol/L.min (moderate dose test) vs. 838.8 ± 34.60 mmol/L.min (high dose test): F (3, 20) = 0.4171: P = 0.0845].

Day 28: There were significant differences in the area under the curve (AUC) values between the four experimental groups [815.0 ± 26.87 mmol/L.min (control) vs. 809.0 ± 7.566 mmol/L.min (low dose test) vs. 917.8 ± 21.44 mmol/L.min (moderate dose test) vs. 945.0 ± 37.68 mmol/L.min (high dose test): F (3, 20) = 1.416: p = 0.0017].

Post-hoc statistical analysis using Tukey’s multiple comparisons test revealed that there were significant differences between the control and the high dose test groups (p = 0.0096), between the low dose test and high dose test groups (p = 0.0067), between the control and moderate dose test groups (p = 0.0481) as well as between the low dose test and moderate dose test groups (p = 0.0341).

Day 42: There were significant differences in the area under the curve (AUC) values between the four experimental groups: [645.0 ± 12.32 mmol/L.min (control) vs. 741.5 ± 16.79 mmol/L.min (low dose test) vs. 796.5 ± 13.21 mmol/L.min (moderate dose test) vs. 921.8 ± 31.93 mmol/L.min (high dose test): F (3, 20) = 1.131: p < 0.0001].

Post-hoc statistical analysis using Tukey’s multiple comparisons test revealed that there were significant differences between the control and the high dose test groups (p < 0.0001), between the low dose test and high dose test groups (p < 0.0001), between the control and moderate dose test groups (p = 0.0002), between the control and low dose test groups (p = 0.0144), as well as between the moderate dose test and high dose test groups (P = 0.0015).

**Fig 2:**
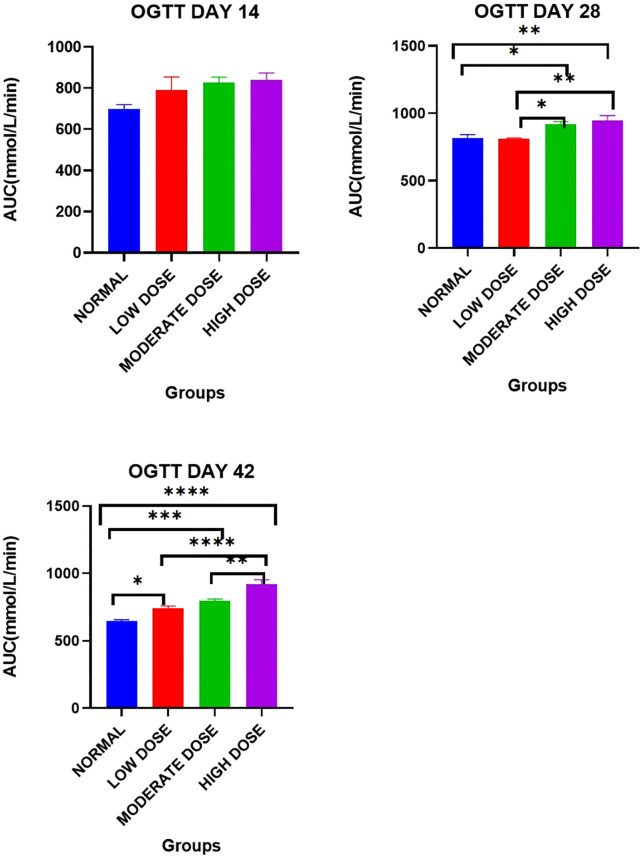
Mean area under the curve (mmol/L.minutes) during the oral glucose tolerance tests. Results are expressed as mean ± SEM. (* p < 0.05, ** p < 0.01, *** p < 0.001, **** p < 0.0001).

The experimental findings are illustrated in Fig 2.

### Effect on adipose tissue distribution

#### Mesenteric adipose tissue mass.

There were significant differences in the mesenteric adipose tissue masses between the four experimental groups on day 42: [0.6595 ± 0.1697 g (control group) vs. 2.082 ± 0.1924 g (low dose test group) vs. 3.079 ± 0.1798 g (moderate dose test group) vs. 3.902 ± 0.2261 g (high dose test group): F (3, 20) = 0.3016: p < 0.0001].

**Fig 3:**
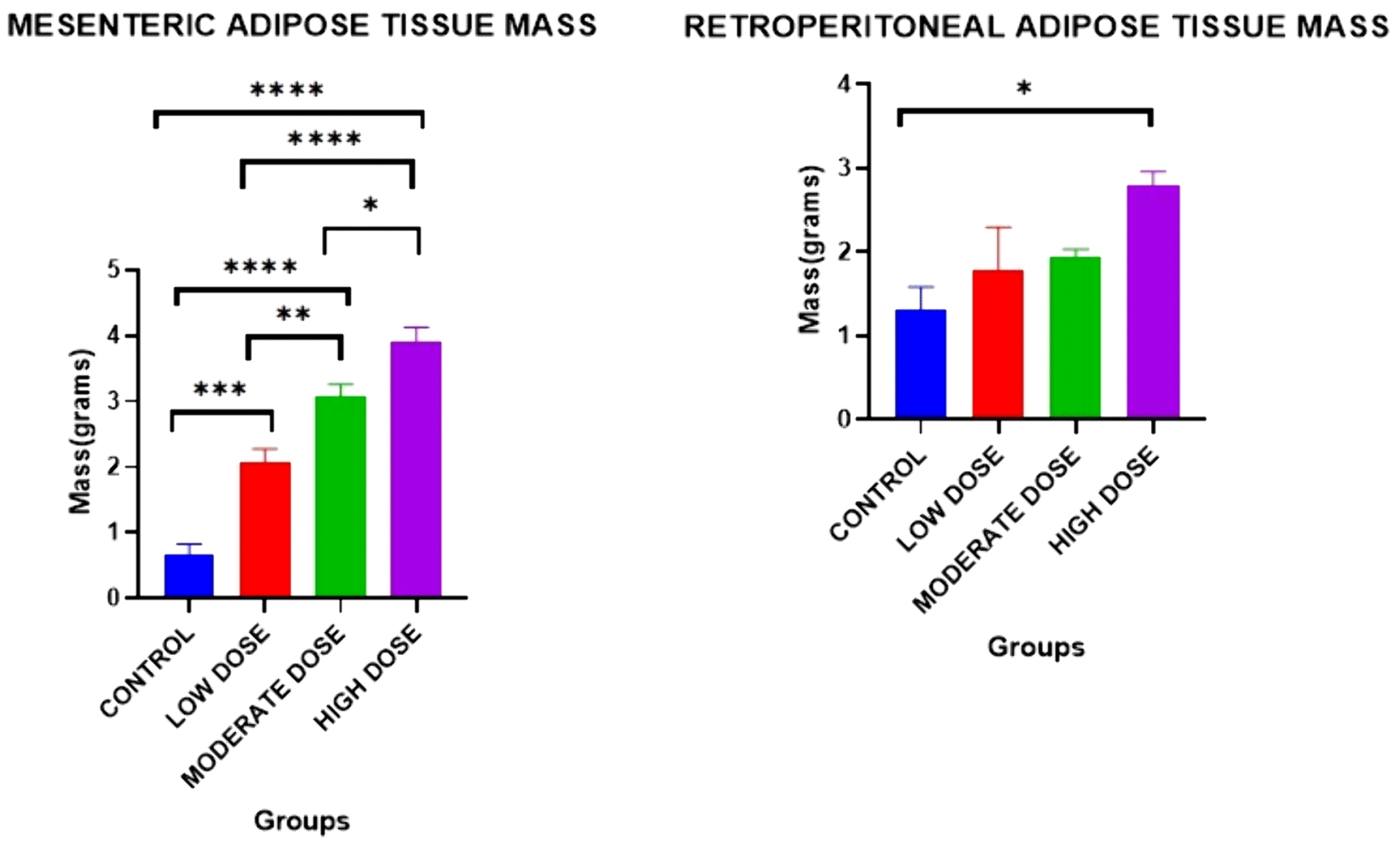
Mean mesenteric adipose tissue mass and mean retroperitoneal adipose tissue mass between all four groups on day 42. Results are expressed as mean ± SEM. (* p < 0.05, ** p < 0.01, *** p < 0.001, **** p < 0.0001).

Post-hoc statistical analysis using Tukey’s multiple comparisons test revealed that there were significant differences were between the control and the high dose test groups (p < 0.0001), between the control and the moderate dose test groups(p < 0.0001), between the low dose test and the high dose test groups (p < 0.0001), between the control and low dose test groups (p = 0.0002), between the low dose test and the moderate dose test groups (p = 0.0080), as well as between the moderate dose test and the high dose test groups (p = 0.0323). The experimental data is illustrated in Fig 3.

#### Retroperitoneal adipose tissue mass.

There were significant differences in the mean retroperitoneal adipose tissue masses between the four experimental groups on day 42: [1.307 ± 0.2743 g (control) vs. 1.778 ± 0.5174 g (low dose test group) vs. 1.936 ± 0.09747 g (moderate dose test group) vs. 2.789 ± 0.1704 g (high dose test group): F (3, 20) = 0.8928: P = 0.002].

Post-hoc statistical analysis using Tukey’s multiple comparisons test revealed that there were significant differences were between the control and high dose test groups (P = 0.0141). The experimental data is illustrated in Fig 3.

There were, however, no significant differences in the mean pericardial adipose tissue mass and mean tongue weights between the four experimental groups on day 42.

### Effect on liver mass

There were significant differences in the liver mass between the four experimental groups on day 42[7.278 ± 0.2837 grams (control group) vs. 8.735 ± 0.7600 grams (low dose test group) vs. 8.877 ± 1.135 grams (moderate dose test group) vs. 11.39 ± 0.6126 grams (high dose test group): F (3, 20) = 5.952: p = 0.0095].

Post-hoc statistical analysis using Tukey’s multiple comparisons test revealed that there were significant differences between control and high dose test groups (p = 0.0055).

### Effect on liver mass/ body mass ratio (hepatic index)

There were significant differences in the hepatic index between the four experimental groups on day 42 [0.03124 ± 0.001857 (control group) vs. 0.03349 ± 0.002972 (low dose test group) vs. 0.03754 ± 0.003061 (moderate dose test group) vs. 0.04666 ± 0.002602 (high dose test group): F (3, 20) = 1.025: p = 0.0030].

**Fig 4:**
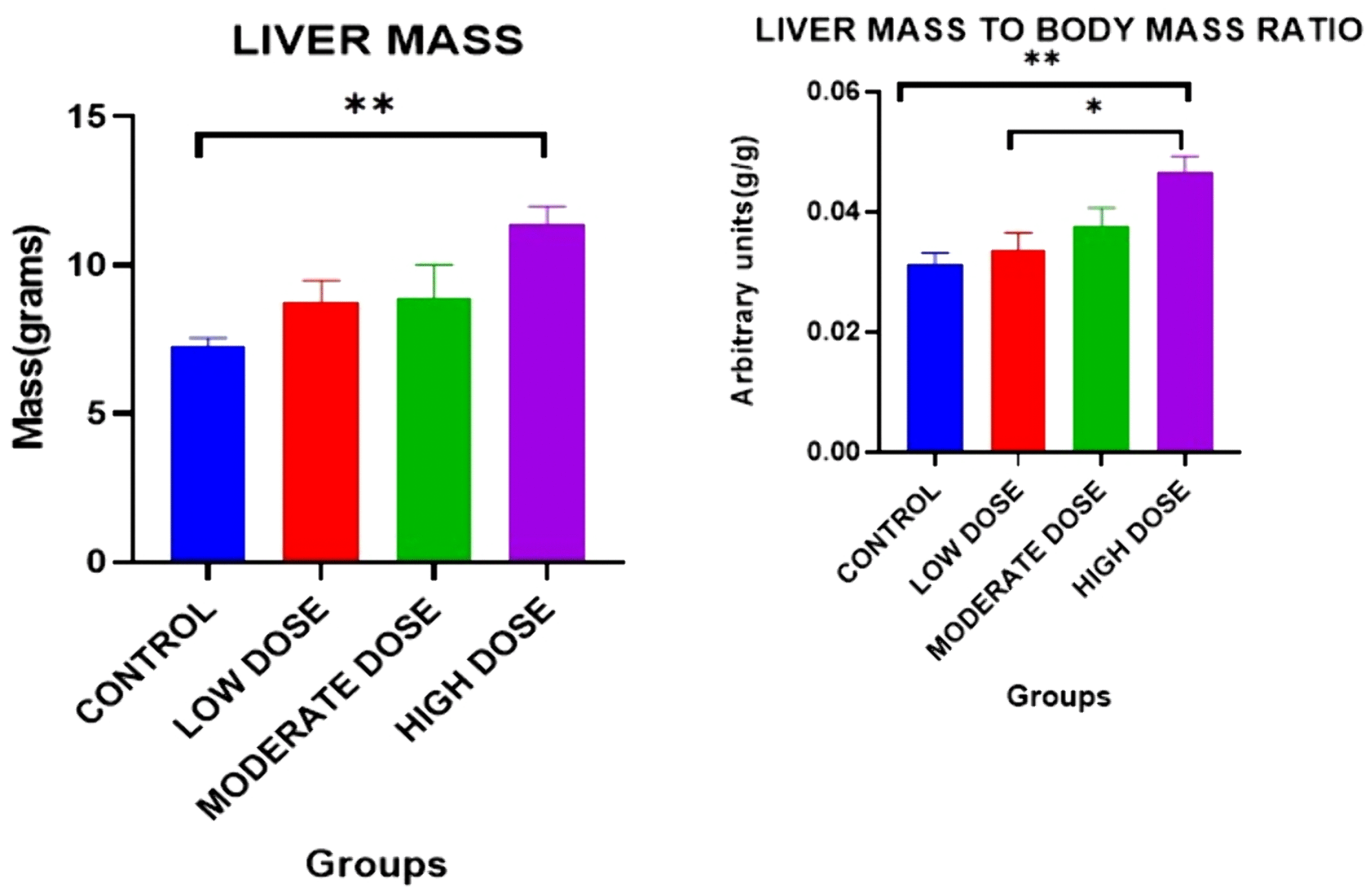
Mean liver mass and mean liver mass: body mass ratios between all four groups on day 42. Results are expressed as mean ± SEM. (* p < 0.05, ** p < 0.01).

Post-hoc statistical analysis using Tukey’s multiple comparisons test revealed that there were statistically significant differences between control and high dose test groups (p = 0.0030), and between the low dose test and high dose test groups (p = 0.0113). The experimental data is illustrated in Fig 4.

### Effect on hepatic triglycerides

There were significant differences in the levels of hepatic triglycerides between the four experimental groups on day 42 [2.177 ± 0.0602 mg/g (control group) vs. 2.475 ± 0.1127 mg/g (low dose test group) vs. 2.736 ± 0.1148 mg/g (moderate dose test group) vs. 3.585 ± 0.1002 mg/g (high dose test group): F (3, 20) = 0.5209: p < 0.0001].

**Fig 5:**
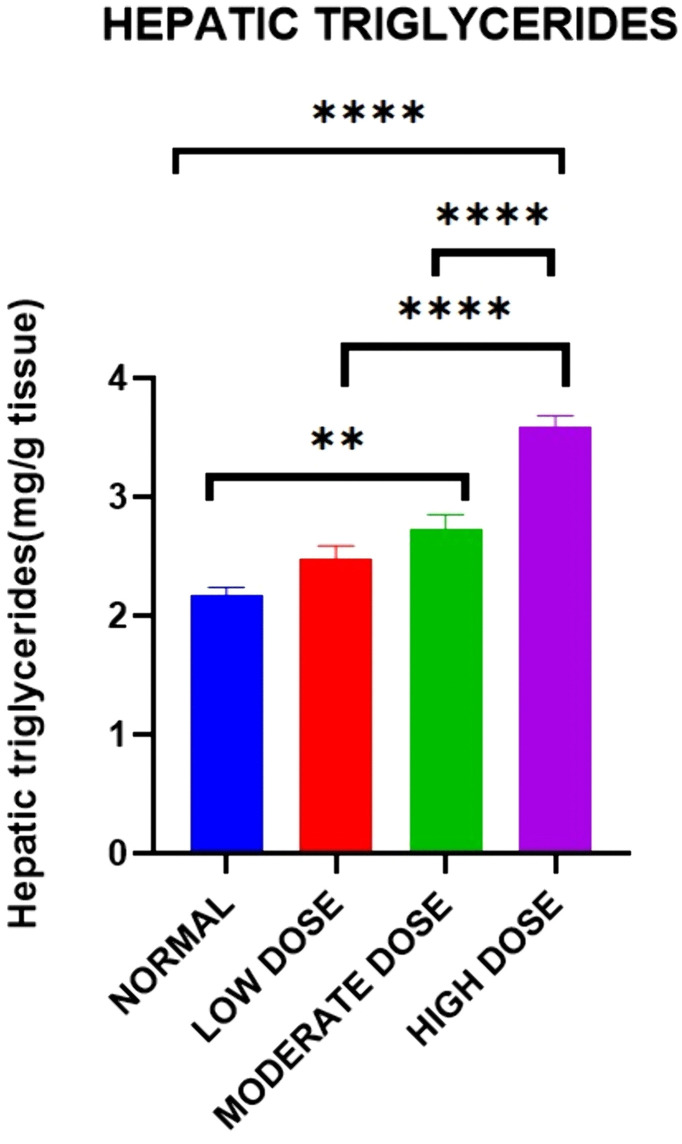
Mean hepatic triglycerides (mg/g) between all four groups on day 42. Results are expressed as mean ± SEM. (* p < 0.05, ** p < 0.01, *** p < 0.001).

Post-hoc statistical analysis using Tukey’s multiple comparisons test revealed that there were significant differences were between the control and the high dose test groups (p < 0.0001), between the low dose test and the high dose test groups (p < 0.0001), between the moderate dose test and high dose test groups (p < 0.0001), as well as between the control and moderate dose test groups (p = 0.0038). The experimental data is illustrated in Fig 5.

### Effect on reductive capacity

There were significant differences in the mean reductive capacity of blood between the four experimental groups on day 42[65894 ± 13487 (control group) vs. 29817 ± 2776 (low dose test group) vs. 17952 ± 3790(moderate dose test group) vs. 9667 ± 2059(high dose test group): F (3, 20) = 0.8928: P = 0.0026].

**Fig 6:**
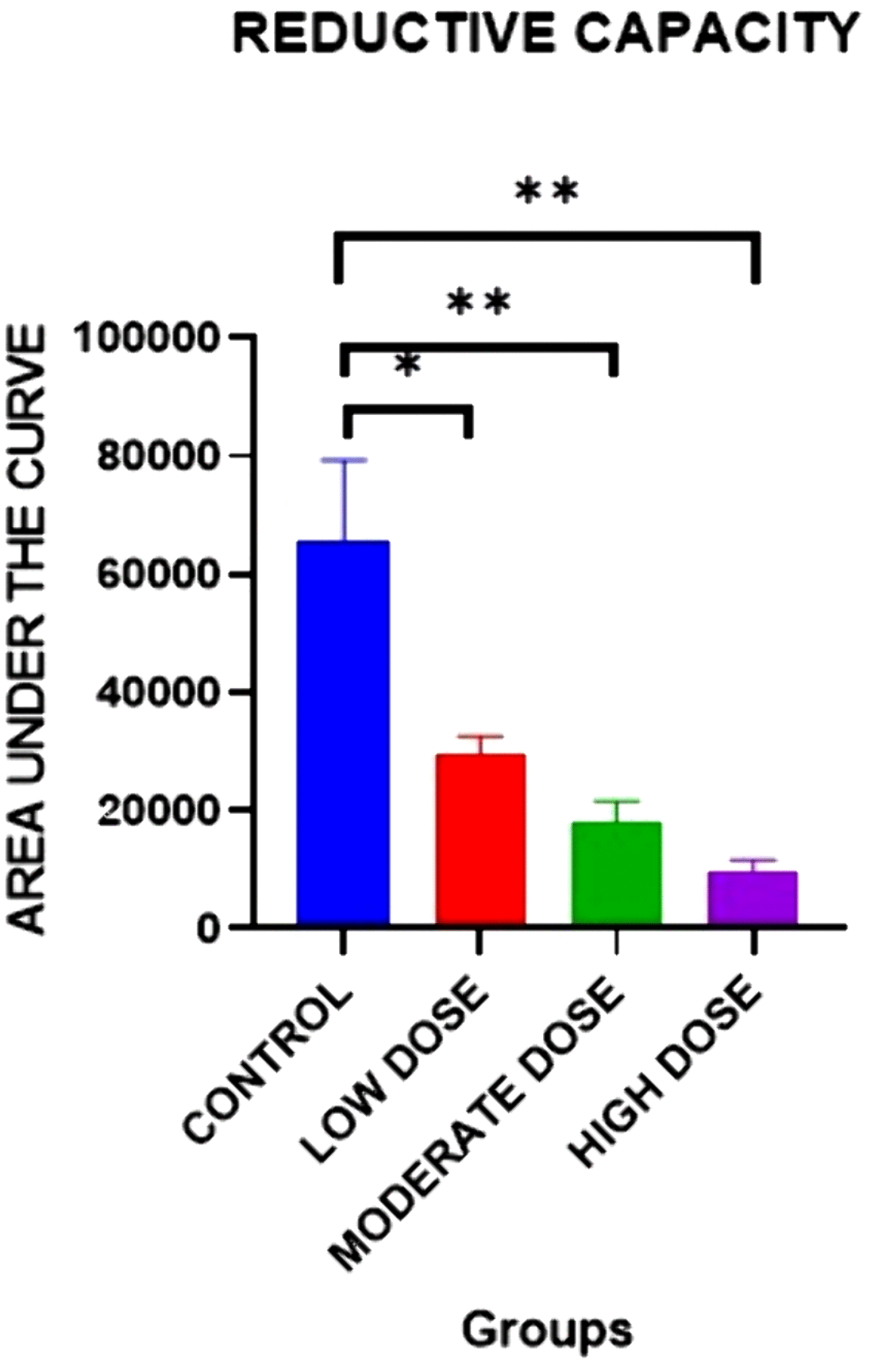
Mean area under the curve showing reductive capacity between all four groups on day 42. Results are expressed as mean ± SEM. (* p < 0.05, ** p < 0.01).

Post-hoc statistical analysis using the Tukey’s multiple comparisons test revealed that there were significant differences were between the control and high dose test groups (P = 0.0025),the control and moderate dose test groups(P = 0.0067), and the control and low dose test groups(P = 0.0314). The experimental data is illustrated in Fig 6.

### Effect on liver function tests

The experimental data is illustrated in Table 1.

**Table 1:** Liver function test results in all four groups on day 42.

Group	Alkaline Phosphatase (ALP) (u/L)	Aspartate Aminotransferase (AST) (u/L)	Alanine Aminotransferase (ALT) (u/L)	Gamma Glutamyl Transpeptidase (GGT) (u/L)
**Control**	29.55 ± 6.307	27.81 ± 4.692	41.50 ± 8.057	2.660 ± 0.8801
**Low Dose Test**	104.4 ± 12.49**	167.3 ± 5.658****	72.67 ± 3.403	7.038 ± 0.8009**
**Moderate Dose Test**	187.7 ± 5.237****	197.7 ± 4.175****	96.33 ± 3.471*	9.173 ± 0.5258****
**High Dose Test**	278.3 ± 24.10****	243.2 ± 9.355****	185.3 ± 24.15****	13.70 ± 0.6788****

Results are expressed as mean ± SEM. (* p < 0.05 vs. control, ** p < 0.01 vs. control, *** p < 0.001 vs. control, **** p < 0.0001 vs. control).

#### Effect on alkaline phosphatase (ALP).

There were statistically significant differences between the four experimental groups in the alkaline phosphatase levels on day 42: [29.55 ± 6.307 u/L (control group) vs. 104.4 ± 12.49 u/L (low dose test group) vs. 187.7 ± 5.237u/L (moderate dose test group) vs. 278.3 ± 24.10 u/L (high dose test group): F (3, 20) =2.194: p < 0.0001].

Post-hoc statistical analysis using Tukey’s multiple comparisons test revealed that there were significant differences between the control and the high dose test groups (p < 0.0001), between the control and the moderate dose test group (p < 0.0001), between the low dose test and the high dose test groups(p < 0.0001), between the moderate dose test and the high dose test groups(p < 0.0011), between the low dose test and the moderate dose test groups (p < 0.0026), as well as between the control and the low dose test groups (p < 0.0066).

#### Effect on aspartate aminotransferase (AST).

There were statistically significant differences between the four experimental groups in the aspartate aminotransferase levels on day 42 [27.81 ± 4.692 u/L (control group) vs. 167.3 ± 5.658 u/L (low dose test group) vs. 197.7 ± 4.175 u/L (moderate dose test group) vs. 243.2 ± 9.355 u/L (high dose test group): F (3, 20) =1.872: p < 0.0001].

Post-hoc statistical analysis using Tukey’s multiple comparisons test revealed that there were statistically significant differences between the control and the high dose test groups (p < 0.0001), between the control and the moderate dose test groups (p < 0.0001), between the control and the low dose test groups(p < 0.0001), between the low dose test and the high dose test groups(p < 0.0001), between the moderate dose test and the high dose test groups (p < 0.0003), as well as between the low dose test and the moderate dose test groups (p < 0.0137).

#### Effect on alanine aminotransferase (ALT).

There were statistically significant differences between the four experimental groups in the alanine aminotransferase levels on day 42 [41.50 ± 8.057 u/L (control group) vs. 72.67 ± 3.403 u/L (low dose test group) vs. 96.33 ± 3.471 u/L (moderate dose test group) vs. 185.3 ± 24.15 u/L (high dose test group): F (3, 20) =3.183: p < 0.0001].

Post-hoc statistical analysis using Tukey’s multiple comparisons test revealed that there were statistically significant differences between the control and the high dose test groups (p < 0.0001), between the low dose test and the high dose test groups (p < 0.0001), between the moderate dose test and the high dose groups (p < 0.0005), as well as between the control and the moderate dose test groups (p < 0.0335).

#### Effect on gamma glutamyl transpeptidase (GGT).

There were statistically significant differences in the gamma glutamyl transpeptidase levels on day 42 [2.660 ± 08801 u/L (control group) vs. 7.038 ± 0.8009 u/L (low dose test group) vs. 9.173 ± 0.5258 u/L (moderate dose test group) vs. 13.70 ± 0.6788 u/L (high dose test group): F (3, 20) =0.1486: p < 0.0001].

Post-hoc statistical analysis using Tukey’s multiple comparisons test revealed that there were statistically significant differences between the control and the high dose test groups (p < 0.0001), between the control and the moderate dose test groups (p < 0.0001), between the low dose test and the high dose test groups (p < 0.0001), between the moderate dose test and the high dose test groups (p < 0.0016), as well the control and the low dose test group (p < 0.0022).

#### Effect on free heme concentration in blood plasma.

There were statistically significant differences in the free heme concentration levels on day 42 [5.221 ± 0.2227 mmol (normal control) vs. 5.887 ± 0.1123 mmol (low dose group) vs. 6.662 ± 0.2264 mmol (moderate dose group) vs. 7.770 ± 0.2175 mmol (high dose group): F (3, 20) =0.4849: p < 0.0001].

**Fig 7:**
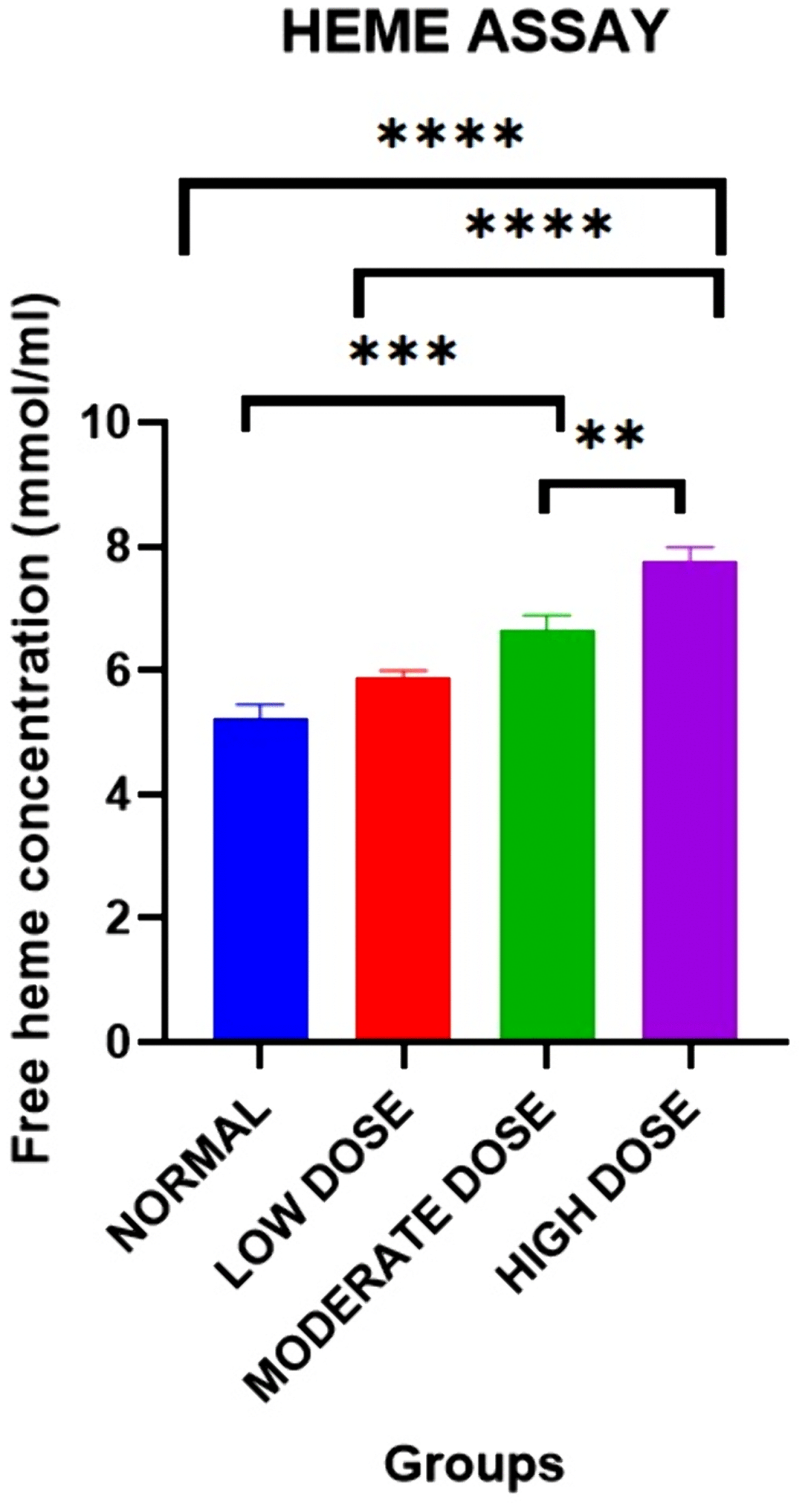
Mean free heme concentration in plasma in all four groups on day 42. Results are expressed as mean ± SEM. (* p < 0.05, ** p < 0.01, *** p < 0.001, **** p < 0.0001).

Post-hoc statistical analysis using Tukey’s multiple comparisons test revealed significant differences were between the normal group and the high dose test group (p < 0.0001), the low dose group and the high dose group (p < 0.0001), the normal group and the moderate test group (p = 0.0003), and the moderate dose group and the high dose group (p = 0.0045). The experimental data is illustrated in Fig 7.

#### Effect on liver histology.

**Fig 8:**
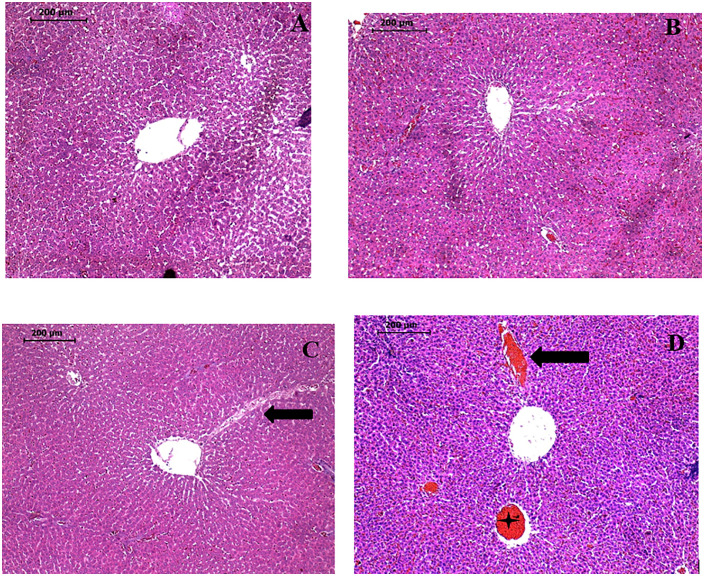
Histology of liver sections of all four groups stained with hematoxylin-eosin staining. **(X 10)** A- Control group- Low dose test group, C- Moderate dose test group and D-High dose test group.

Histological differences were observed in the liver tissue of the experimental groups. Central vein congestion in acetylene-exposed groups as shown by the dilated central veins and dilated sinusoids (shown by black arrows).Central vein obstruction in the high dose group (shown by asterisk). This is illustrated in Fig 8.

## Discussion

The globalization of agricultural trade which is accompanied by the integration of developing country agricultural producers into global supply chains has in turn resulted in all year demand for tropical fruits leading to an increase in the use of artificial ripening of fruits [19,20]. Indeed, it has been estimated that nearly 70–80% of fruits in developing countries, are artificially ripened using different agents, underscoring the potential health hazards associated with these practices [21]. The foregoing discussion highlights the need to conduct basic and applied research to evaluate the short- as well as long- term health effects on farmers, traders and even end-consumers of these ripening practices [21]. This study investigated the cardiometabolic effects of sub-chronic exposure to crude acetylene generated from calcium carbide- a ripening agent whose popularity is increasing rapidly [22]. While ingestion remains the main route of consumer exposure to calcium carbide-ripened fruits, our study deliberately focused on inhalation to model occupational risk. In many low- and middle-income country settings, fruits are ripened in small, poorly ventilated rooms where acetylene gas accumulates, exposing workers and nearby individuals for prolonged periods. The expansion of commercial agriculture in regions such as sub-Saharan Africa further heightens this risk. For example, Kenya, which is now Africa’s largest producer of avocados, has experienced a surge in fruit handling and postharvest operations that commonly employ crude acetylene for ripening [23]. This growing commercialization increases the number of workers repeatedly exposed to acetylene in enclosed ripening facility posing occupational health risks. Our findings therefore fill a critical gap by highlighting inhalation as a major but under-recognized pathway of exposure with significant public health implications.

Crude acetylene did not have any significant effects on body weight in this study. These results are similar to those reported in literature where acetylene was shown not to have any effects on body weight [8]. However, it is noteworthy that the crude acetylene caused significant increases in retroperitoneal and mesenteric adipose tissue mass indicating that it was causing an increase in central adiposity. This to our knowledge is a novel and intriguing finding. This because numerous animal and human studies have reported that central adiposity even in the presence of normal BMI or normal body weight is associated with greater than 3-fold higher risk of all-cause mortality and/or cardiovascular events than in matched controls without the associated changes in adipose tissue distribution [24–26].

Crude Acetylene had significant deleterious dose-dependent effects on glycemic control as shown by its effects in the fasting blood glucose and OGTT experiments between the four groups during the experimental period. These findings are to our knowledge novel. The pathophysiology of elevated fasting blood glucose is known to be associated with increased hepatic insulin resistance, pancreatic ß-cell dysfunction, or chronic low ß-cell mass, deranged glucagon-like peptide-1 secretion, and glucagon hypersecretion among others [27–29]. The pathophysiology of impaired glucose tolerance on the other hand has been shown to be characterized by heightened peripheral insulin resistance, normal hepatic insulin sensitivity, progressive ß-cell dysfunction, reduced secretion of the insulin-tropic hormones, and deranged glucagon secretion [27,30–33]. In addition, patients with combined elevated fasting glucose and impaired glucose tolerance exhibit severe defects in both peripheral and hepatic insulin sensitivity as well as a progressive loss of β-cell function [28] It appears therefore that crude acetylene negatively affects glycemic control via multiple pathophysiological mechanisms.

There were significant differences in the hepatic triglyceride levels between the experimental groups. Exposure to crude acetylene resulted in dose-dependent increases in hepatic triglyceride levels which are characteristic of Metabolic (dysfunction)-associated fatty liver disease [34]. Metabolic (dysfunction)-associated fatty liver disease (MAFLD) which is a condition that affects a third of the global population and whose prevalence has increased in parallel with and is associated with type 2 diabetes mellitus (T2DM), dyslipidemia, obesity, which are features of the metabolic syndrome [35]. It has been shown to increase the risk of end-stage liver disease, hepatocellular carcinoma, and death in the absence of liver transplantation as well as other extrahepatic consequences, including but not limited cardiometabolic disease and cancers [36].

There were significant dose dependent elevations in the levels of the liver enzymes assayed, i.e., GGT, ALT, ALP, and AST. These findings are similar to those in literature where elevations in AST, ALT, ALP and GGT were recorded [5,37]. It is well established that elevations in the ALP and AST levels indicate that the liver damage is hepatocellular in nature while elevations in the levels of GGT and ALP indicate cholestatic damage [38]. Indeed, exposure to environmental chemicals has emerged as a significant contributor to liver disease, including NAFLD with Alanine aminotransferase (ALT) in particular, being considered a specific biomarker of liver injury and is widely used in epidemiological studies [39,40]. On the other hand, GGT which is an enzyme located on the external surface of cellular membranes whose increased activity is a marker of antioxidant inadequacy and increased oxidative stress [41]. In addition, Previous studies have shown that GGT is a sensitive marker of insulin resistance in adults with a close association between metabolic dysregulation, steatosis degree and GGT levels in patients with NAFLD being reported [42–44]. Further, it has been previously shown that the increase in GGT levels is closely correlated to the degree of steatosis increased, and most importantly normalization of GGT levels has been proposed as a predictor of histological improvement, improved metabolic control, and especially inflammation, in the routine management of patients with NAFLD [43–45].

The foregoing discussion underscores the canonical mechanistic role played by insulin resistance in stimulating hepatic de novo lipid synthesis, which in turn result in the increased release of fatty acids and derived products, such as triglycerides and cholesterol observed in MAFLD [42,46]. In addition, the results of the glycemic control, hepatic triglyceride and liver function tests indicate that exposure to crude acetylene resulted in the generation of a MAFLD-like syndrome which however occurred within a background of normal BMI but with increased central adiposity. This to our knowledge has not been previously reported in literature and is therefore a novel finding. Although MAFLD is usually clinically comorbid with obesity, it is also observed in a significant proportion of lean patients, especially Asians [36]. Notably, individuals with lean MASLD face equal or higher overall mortality rates compared to their non-lean counterparts with the former being associated with an increased risk of Hepatocellular Carcinoma, while their non-lean counterparts are more prone to cardiovascular outcomes and T2DM [35,36]

Acetylene exposure led to significant dose- dependent reductions in the mean reductive capacity of blood assayed using the nitrocellulose redox permanganometry (NRP) technique [15]. Although this is the first time to our knowledge that the changes in blood reductive capacity in response to acetylene exposure are being reported, these results are in broad agreement with those of a previous study where the serum SOD activity, GSH level, and FRAP were significantly decreased on acetylene exposure indicating increased oxidative stress [37]. Increased oxidative stress has been previously shown to compromise insulin signaling via several ways including but not limited to, activation of the NF-κB pathway via the phosphorylation of its inhibitory subunit, IκB, by the serine kinase IKK, disruption of the intracellular distribution of PI3K, activation of serine/threonine kinases which then phosphorylate the serine/threonine residues of components of the insulin signaling pathway such as the Insulin Receptor and Insulin Receptor Substrate elevating the risk of insulin resistance and diabetes mellitus [47,48]. In addition, oxidative stress has been shown to cause a reduction in GLUT4 expression and promote apoptosis of the beta cells [48–50].

Exposure to crude acetylene resulted in significant dose-dependent increases in free heme concentrations in plasma. This finding to our knowledge is a novel one which suggests that exposure to crude acetylene led to increased intravascular hemolysis. Previous studies have shown that crude acetylene exposure as well as administration of Calcium Carbide were associated with deleterious effects on the following red blood cell indices: Hb, PCV, RBC, MCV, MCH, MCHC and PLT indicating increases in rates of hemolysis and/or hematopoiesis [5,37,51]. Accumulation of excess free heme the in blood stream which overwhelms the body’s heme scavenging systems leads to several deleterious effects [52]. Heme is the best characterized Hemoglobin-derived Damage associated molecular pattern (DAMP)/alarmin that targets different immune and non-immune cells [52]. It activates Toll-like receptor 4 (TLR4) signaling resulting in the triggering of oxido-inflammatory and thrombotic events by inducing the activation of platelets, endothelial and innate cells as well as the coagulation and complement cascades [53,54]. Its ability to promote sterile inflammation via inflammatory cytokine release and impired resolution of tissue damage [55]. Indeed, heme has been shown to generate a cytokine storm associated with significant increases in the plasma levels of 19 cytokines, including IL-1b, IL-2, IL-6, IL-9, and TNF-α [54–56]. The foregoing discussion therefore provides a putative mechanism for the oxidative stress and inflammation observed in crude acetylene exposure and calcium carbide administration [5,37,51].

Histological examination revealed central vein congestion, evidenced by the dilated central veins, suggesting impaired blood flow and potential venous obstruction, a common reaction to toxic exposure [57].This could be as a result of the oxidative stress from crude acetylene inhalation damaging the endothelial cells that line blood vessels impairing blood flow within the liver’s microcirculation, eventually causing congestion in the central veins [5].Also, the hemolysis as a result of crude acetylene exposure releases free hemoglobin into the bloodstream, placing additional strain on the liver as it processes the excess hemoglobin and its byproducts. As a result, central vein congestion and sinusoidal dilation may reflect the liver’s efforts to cope with both impaired blood flow and the added burden of hemolysis.

In conclusion, the results of this study indicate that the sub chronic exposure to crude acetylene resulted in derangements in secondary metabolism that manifested in poor glycemic control, central adiposity and hepatic steatosis which were manifested against a background of normal BMI and body weight. These metabolic derangements were accompanied by a decreased plasma reductive capacity/increased oxidative stress and increased levels of free heme. These results highlight the severe health risks associated with crude acetylene exposure and underscore the need to implement strict regulations to limit the use of calcium carbide in fruit ripening, particularly in occupational settings. The results of this study add to the burgeoning literature on the role of occupational factors in driving the increased rates of metabolic syndrome in Sub Saharan Africa and other LMICs.

While this study provides valuable preliminary insights into the metabolic effects of sub-chronic crude acetylene exposure, several limitations should be acknowledged. One methodological consideration of this study is the type of inhalation chamber used. We employed a whole-body chamber with inlet and outlet ports, allowing regular replenishment of crude acetylene to maintain stable exposure conditions. As much as this approach ensured reproducibility, it does not provide the same level of precision as advanced flow-through inhalation systems that allow automated continuous regulation of airflow and gas composition. Future studies could therefore build on our findings by using such systems to better simulate real-world occupational exposures. Also notably, the absence of mechanistic assays such as the Insulin Tolerance Test (ITT), insulin measurements or HOMA-IR calculations limits the ability to fully characterize insulin sensitivity and delineate the underlying metabolic pathways. Incorporating these assessments in future studies would enhance mechanistic understanding and strengthen the current findings.

Additionally, while elevated hepatic triglyceride levels suggest hepatic steatosis biochemically, the lack of histological confirmation through Oil Red O staining is a limitation. Future research should include such staining to provide visual evidence of lipid accumulation in liver tissue. The study also did not measure daily food intake, which could influence metabolic parameters. Monitoring dietary intake in subsequent studies would help control for this potential confounder.

Future investigations should also explore potential therapeutic strategies to address the complex interplay between red blood cell hemolysis, oxidative stress, and inflammation. In particular, understanding the multidirectional relationships among these factors and the inter-organ cross-talk between the liver, adipose tissue, and blood cellular elements will be crucial in developing effective interventions.

## Supporting information

S1 File Gatwiris data all. (XLSX)
